# Effect of acute high-intensity interval exercise on a mouse model of doxorubicin-induced cardiotoxicity: a pilot study

**DOI:** 10.1186/s13102-024-00881-x

**Published:** 2024-04-26

**Authors:** Elise P. Legault, Paula A. B. Ribeiro, Daniil R. Petrenyov, Gergana O. Drumeva, Charles Leduc, Sharmila Khullar, Jean N. DaSilva, Alain Steve Comtois, François B. Tournoux

**Affiliations:** 1 @coeurlab research unit, Centre de recherche du Centre Hospitalier de l’Université de Montréal, 900 St Denis Street, Montréal, Québec Canada; 2 Département des sciences de l’activité physique, Université du Québec à Montréal, Montréal, Québec Canada; 3 Département de pharmacologie et physiologie, Université de Montréal, Montréal, Québec Canada; 4 Département de pathologie et biologie cellulaire de l’Université de Montréal, Montréal, Québec Canada; 5 Département de radiologie, radio-oncologie et médecine nucléaire, Université de Montréal, Montréal, Québec Canada; 6 Service de Cardiologie du Centre Hospitalier de l’Université de Montréal, Montréal, Québec Canada

**Keywords:** Echocardiography, High-intensity interval training, Hypertrophy, Heart failure, Breast cancer

## Abstract

**Background:**

It is unknown whether high-intensity interval exercise (HIIE) may potentiate or attenuate the cardiotoxic effect of chemotherapy agents such as doxorubicin (DOX) when performed shortly after treatment. The study aimed to investigate the effect of acute HIIE on cardiac function and structure performed either 1, 2 or 3 days after DOX injection in an animal model.

**Methods:**

Female C57bl/6 mice (*n* = 28), 70 days old, received a bolus 20 mg/kg intravenous tail vein DOX injection. Three exercise groups performed 1 HIIE session (16 sets of 1 min at 85–90% of peak running speed) at 1 (*n* = 7), 2 (*n* = 7), and 3 days (*n* = 8) following the DOX injection. A sedentary (SED) group of mice (*n* = 6) did not exercise. Animals underwent echocardiography under light anesthesia (isoflurane 0.5-1%) before and 7 days after the DOX injection. Animals were sacrificed on day 9 and hearts were collected for morphometric and histological analysis.

**Results:**

Animals exercising on day 3 had the smallest pre-post reduction in left ventricular fractional shortening (LVFS) (MΔ= -1.7 ± 3.3; *p* = 0.406) and the SED group had the largest reduction (MΔ=-6.8 ± 7.5; *p* = 0.009). After reclassification of animals according to their exercise compliance (performing > 8/16 of high-intensity bouts), LVFS in compliant mice was unchanged over time (LVFS MΔ= -1.3 ± 5.6; *p* = 0.396) while non-compliant animals had a LVFS reduction similar to sedentary animals. There were no significant differences in myocardial histology between groups.

**Conclusions:**

In this pilot murine study, one single HIIE session did not exacerbate acute doxorubicin-induced cardiotoxicity. The timing of the HIIE session following DOX injection and the level of compliance to exercise could influence the negative impact of DOX on cardiac function.

**Supplementary Information:**

The online version contains supplementary material available at 10.1186/s13102-024-00881-x.

## Background

Anthracyclines including doxorubicin (DOX) and epirubicin are commonly used to treat various malignant tumors such as breast cancer, leukemia, lymphomas and sarcomas [1–3]. One of the main side effects of anthracyclines is myocardial toxicity, which can lead to clinical heart failure [4, 5]. The pathogenesis of DOX-induced cardiotoxicity is still being investigated; among the main proposed mechanisms is an increase of reactive oxygen species (ROS) leading to lipid peroxidation and membrane damage accompanied by an activation of caspase and DNA degradation triggering cardiomyocyte apoptotic pathways [6].

Exercise training during cancer treatment can effectively improve cardiopulmonary fitness, quality of life and relieve treatment related symptoms [7–9]. Furthermore, studies have shown that exercise training during anthracycline treatment is feasible and attenuates cardiac dysfunction in breast cancer patients [10, 11]. Both acute exercise and chronic training have demonstrated cardioprotective effects in animal models [12, 13]. Previous studies showed that an acute bout of moderate-intensity exercise performed 24 h prior to treatment in murine models attenuated DOX related cardiac dysfunction, cardiomyocyte mitochondrial function and reduced cardiac oxidative burden by reducing ROS [13, 14]. Most studies have focused on the cardioprotective effects of moderate intensity exercise or voluntary free wheel exercise prior and during anthracycline [12], but very few have investigated the effect of high-intensity exercise, especially in acute models. High-intensity interval training (HIIT) consists of short, repeated bouts of near maximal physical effort (85–95% of maximal aerobic capacity) alternating with low intensity effort [15, 16]. This type of training has greater efficiency than continuous exercise for improving cardiorespiratory capacity (VO_2_) and function in patients with heart failure and coronary diseases [17–20]. However, efficacy of this type of exercise performed concomitantly with anthracycline treatment remains unclear. Also, HIIT could potentiate cardiotoxicity, especially if performed shortly after DOX treatment, since exercise acutely increases ROS production [21], and the magnitude of this phenomenon is modulated by exercise duration and intensity.

Therefore, the purpose of this study was to investigate the effect of HIIE on cardiac function and structure performed either 1, 2 or 3 days after DOX injection in an acute cardiotoxicity mouse model. The main outcome measure was left ventricular fractional shortening (LVFS) measured by echocardiography as a marker of cardiac function. Secondary outcome measures were morphological and histopathological signs of cardiac remodeling.

## Methods

### Animals

This study is reported in accordance with the *Animals in Research: Reporting In Vivo Experiments* (ARRIVE) guidelines [22]. All procedures were approved by the institutional committee for the protection of animals (CIPA, protocol number: CM18001FTs) of the University of Montreal Hospital Research Center (CRCHUM, Montreal, Canada). Animals received humane care in compliance with the Canadian Council on Animal Care and the *Guide for the Care and Use of Laboratory Animals* published by the US National Institutes of Health (NIH Publication No. 85 − 23, revised 1985). Female C57bl/6 mice (*n* = 30) from Charles River Laboratories (Saint-Constant, Quebec, Canada), 64–70 days old, mean body mass of 17.9 g ± 1.1 g (SD) upon arrival, were acclimated to 12:12-hour artificial light-dark cycles at the research facility, 48 h before the start of the protocol. Animals were housed 3 to 5 mice per cage in an enriched environment, fed on a regular chow diet and had access to water ad libitum. Female mice were chosen since previous studies have shown that adult male rodents are more sensitive to the cardiotoxic effect of doxorubicin than females [23] and have a lower survival rate after a bolus injection of high dose DOX [24]. Our group showed that female mice are less lethargic and therefore probably more willing to exercise after DOX (supplementary file 1). Mice were randomly assigned to one of the following groups: sedentary (SED; *n* = 7), exercise groups consisting of one HIIE session performed either 1 day (G1; *n* = 7), 2 days (G2; *n* = 8), or 3 days (G3; *n* = 8) post-DOX. Treatment allocation was determined using a computerized random list tool (https://www.randomizer.org/).

### Treatment

All animals received a single 20 mg/kg intravenous tail vein bolus injection of DOX (hydrochloride ≥ 98%, Cayman Chemical, CO) dissolved in saline. This bolus dose was chosen considering that it is known to cause a significant reduction in left ventricular fractional shortening (LVFS) within 5 days following injection [25] that occurs much earlier than repeated injections of equivalent cumulative doses but results in similar overall cardiac dysfunction [26]. Following DOX-injection, water drenched chow was placed in the bottom of the cages and renewed daily to encourage animals to eat and stay hydrated to prevent asthenia.

### Exercise session

Treadmill (AccuPacer, Omnitech Electronics, Columbus, Ohio, USA) acclimation protocol and maximal exercise testing were adapted from the study of Petrosino et al. [27]. The electrical stimulus was substituted by gentle encouragement from a human operator using a tongue depressor to slightly lift the mice’s hind legs as previously described [28]. Acclimation was performed three times with 48-60 h of recovery between training sessions. Animals were placed on the static treadmill for 3 min prior to exercise. Initial running speed was set at 6 m/min for 5 min, followed by 9 m/min for 2 min and 12 m/min for the last 2 min. For the entire exercise session, the slope was set to 0 degree [29]. Following acclimation, all mice underwent exercise testing to determine peak running speed (Vpeak), characterized as the greatest speed reached prior to exhaustion, that is when the mice could no longer continue despite repeated encouragement. Each animal’s HIIE running speed was calculated from its individual Vpeak.

The HIIE session started with a 3-minute warm-up (< 50% of Vpeak) followed by 8 sets of 1 min at high-intensity (85–90% of Vpeak). Each set was interrupted by 1 min of active recovery period at low intensity (< 50% of Vpeak). A second series of 8 high-intensity exercise bouts was performed following 5 min of passive recovery, thus reaching a total of 16 high-intensity bouts. The exercise session lasted 40 min or until exhaustion and was performed at a fixed slope of 15 degrees. One HIIE session was performed in the early afternoon either on day 1 (24 h), day 2 (48 h), or day 3 (72 h) following the DOX injection according to each assigned exercise group. Mice who were randomized to the sedentary group did not perform any exercise but were placed in the treadmill cages for 40 min ensuring that all groups were exposed to comparable environmental stress.

### Experimental procedures

**Fig. 1:**
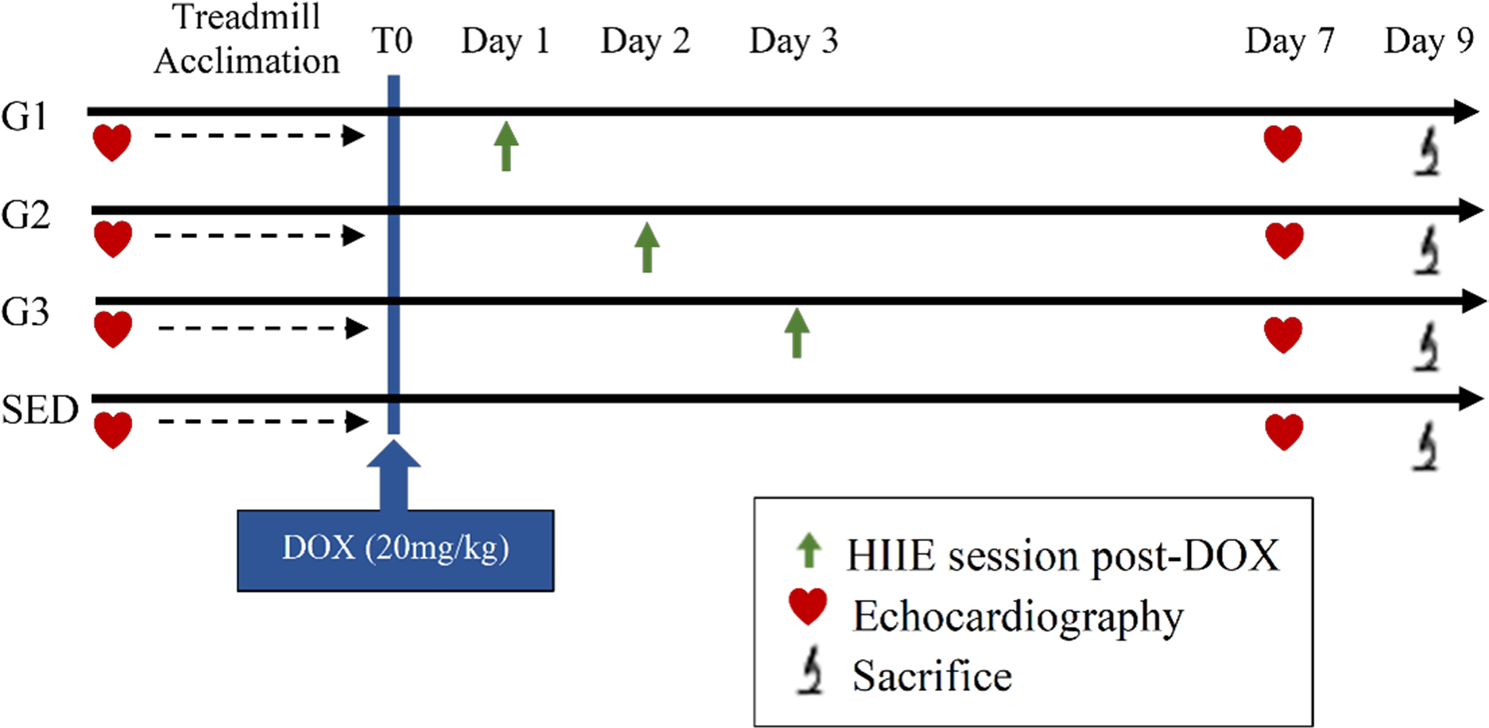
General schematic of the study design and timeline After baseline echocardiography and treadmill acclimation, animals were randomly allocated to one of the four experimental groups: G1, HIIE 1 day following DOX injection; G2, HIIE 2 days following DOX injection; G3, HIIE 3 days following DOX injection; SED, sedentary (no access to exercise). T0, bolus DOX injection; endpoint echocardiography was performed on day 7 following DOX injection and all animals were sacrificed on day 9. DOX, doxorubicin; HIIE, high-intensity interval exercise

All animals were sacrificed on day 9 post-DOX or earlier if they reached ≥ 2 of any of the following intervention endpoints: body mass loss ≥ 20%, asthenia, dehydration, respiratory distress, or severe heart failure observed by echocardiography. Figure 1 depicts the experimental procedure timeline.

### Echocardiography

Transthoracic echocardiography (Vivid E9, GE healthcare; with pediatric transducer i13L, GE, USA) was conducted in lightly anesthetized mice at baseline and 7 days after DOX injection. The anterior thoracic region was shaved on a separate occasion prior to the beginning of the echocardiography to reduce the stress and duration of sedation. Images were obtained in M-Mode from the parasternal short axis view, 5–10 min after a 5-minute isoflurane induction period (2% isoflurane and 0.5 L/min O_2_). To control for the hemodynamic depressive effect elicited by isoflurane, minimal doses (0.5-1% isoflurane and 0.5 L/min O_2_) were used when images were taken, and heart rate was closely monitored to obtain comparable pre- and post-DOX echocardiographic measures that are within normal physiological range for mice [30]. Images were taken and analyzed offline (Echopac7-003781 10.52.1.214, GE healthcare, USA) by an echocardiogram expert (FT) blinded to assigned group. Standardized measurement techniques were used, and mean heart rate (HR) was averaged from three consecutive cycles. Measurements of cardiac dimensions included: LV septal wall thickness and posterior wall thickness, LV end-diastolic diameter and LV end-systolic diameter. Diameters were used to calculate LVFS. LV mass was estimated using Penn’s algorithm [31] as described in supplementary file 3.

### Necropsy

Animals were weighed, anesthetized with 3% isoflurane; 0.5 L/min O_2_ and euthanized by exsanguination from the inferior vena cava followed by a pleural puncture. Hearts were excised, stripped of fat, rinsed in ice-cold PBS, lightly blotted to remove excess blood; then weighted and longitudinally bisected from the apex along the left atrium. Half of the heart was embedded in OCT (Optimal Cutting Temperature compound) and immediately frozen in isopentane at -80 °C for future analysis; the other half was fixed for histological examination in 10% neutral buffered formalin for 24–48 h and embedded in paraffin. The left leg was severed above the knee joint and mechanically stripped to the bone. Tibia length was measured from the condyles to the tip of the medial malleolus using a micrometer caliper (Vernier caliper, Mitutoyo, Co., Japan). Tibia length was used to normalise heart mass for comparison, as previously described [32].

### Histopathologic examination

The paraffin heart tissues were sectioned at a thickness of 5 µm and stained with hematoxylin and eosin in accordance with standard procedures. Two trained pathologists blinded to the group assignment examined the tissue sections under light microscopy to quantify necrosis, cytoplasmic clearing, vacuolisation, inflammation, fibrosis and cardiomyocyte hypertrophy. Histological scores of cardiomyocyte hypertrophy ranged from 0 to 3, with 0 representing an absence of cell hypertrophy; 1 ≤ 25%; 2 between 25 and 50%; and 3 > 50% of cells with hypertrophic changes.

### Data processing and statistical analysis

Data were analyzed using IBM’s SPSS Statistics® (version 26.0; SPSS Inc., Chicago IL, USA) and GraphPad Prism® (version 8.4.2; San Diego, CA, USA) was used to produce graphs. Outcome measures were tested for normality through visual inspection of normality plots and analysis of skewness and kurtosis. Data are reported as group means (M) and standard deviations (SD), or medians (Md) and interquartile intervals (IQR) for non-normally distributed data. Statistical analysis of LVFS pre- and post-DOX was conducted using a repeated measures ANOVA and Bonferroni corrected post hoc test when appropriate. Pairwise comparisons of LVFS pre- and post-DOX injection were computed as mean deltas (MΔ) and bias-corrected accelerated bootstrap 95% confidence intervals (CI). Cohen’s *d* effect sizes were also calculated. Group comparisons were conducted using the Kruskal-Wallis test for non-parametric continuous variables with the Dunn-Bonferroni corrected post hoc test if appropriate and *Fisher’s exact test* for categorical variables. Significance was determined at a *P*-value of ≤ 0.05 for all statistical analyses.

Considering that some of the mice would have been unable or unwilling to complete the training session, additional analyses were performed by merging the exercise groups and allocating the mice according to their exercise session compliance to further explore the safety of this intervention. For analytical purposes, mice were allocated to the compliant (C) group if they performed more than 50% (> 8/16 bouts), and in the non-compliant (N-C) group if they performed less than or equal to 50% of total HIIE session.

## Results

### HIIE-DOX animal model

Two mice were excluded from the final analysis: one mouse from the SED group died unexpectedly, immediately prior to the DOX injection, and one mouse from the G2 group developed pyelonephritis (diagnosed on necropsy) and was euthanized. Therefore, *n* = 28 mice were included in the final analyses (SED *n* = 6; G1 *n* = 7; G2 *n* = 7, and G3 *n* = 8). None of the mice refused to run at baseline during the acclimation period and the peak exercise testing. Mean Vpeak was 37.2 ± 4.9 m/min, average running speed for high-intensity bouts was 33.5 ± 4.4 m/min and 18.6 ± 2.5 m/min for active recovery between bouts. The overall post-DOX compliance rate for the HIIE groups, defined as > 8 high-intensity bouts completed of the 16 bouts prescribed, was 54%. Figure S2.1 shows individual and median number of bouts completed according to group (supplementary file 2). No significant difference in exercise compliance was found between G1 (*n* = 3; 43% compliant), G2 (*n* = 4; 57% compliant, or G3 (*n* = 5; 62,5% compliant) exercise groups (*p* = 0.867; *Fisher’s exact test*). Two mice from the G2 exercise group were euthanized at the end of day 8 because they had reached ≥ 2 of the study’s intervention points for ethical reason (body mass loss ≥ 20%, severe asthenia, and dehydration).

### HIIE effect according to the timing of exercise following DOX

**Fig. 2:**
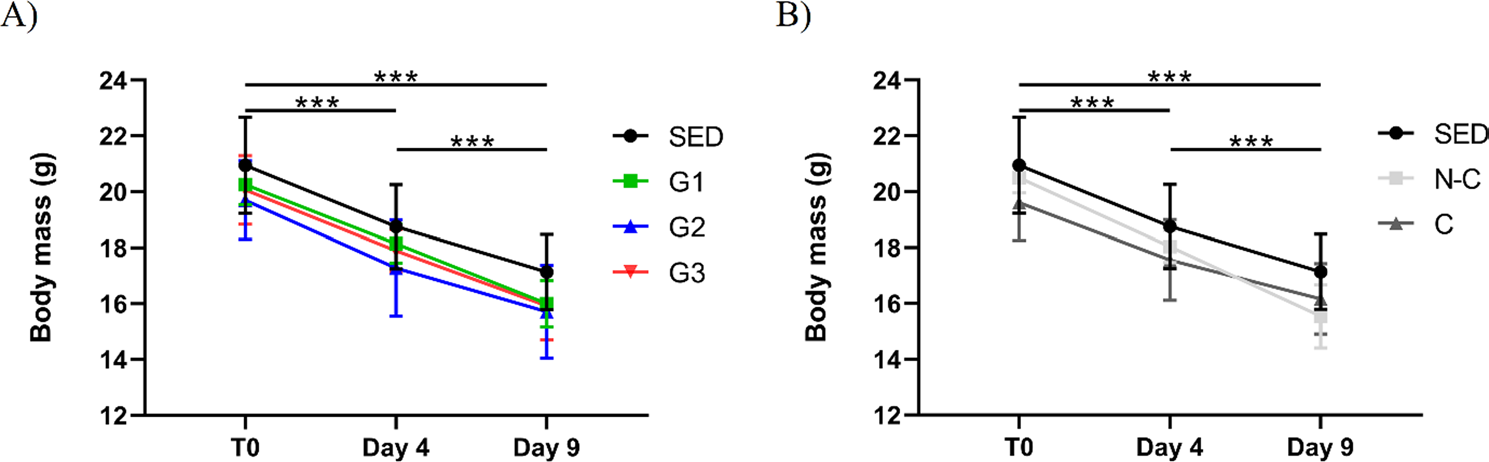
Body mass following doxorubicin injection (**A**) according to intervention groups, there is a significant time effect (*p* < 0.001); (**B**) according to exercise compliance, there is a significant (*p* = 0.018) time*compliance interaction and a significant time effect (*p* < 0.001). ***P < 0.001, represents repeated measures ANOVA Bonferroni corrected post hoc for time effect. Values are mean ± SD. C, compliant (*n* = 12); G1, HIIE day 1 post-DOX (*n* = 7); G2, HIIE day 2 post-DOX (*n* = 7); G3, HIIE day 3 post-DOX (*n* = 8); N-C, non-compliant (*n* = 10) and SED, no exercise (*n* = 6)

*Effect on body mass.* Body mass according to the experimental group is presented in Fig. 2A and body mass deltas between baseline and endpoint were SED= -3.8 ± 0.8 g; G1= -4.3 ± 1.3 g; G2= -4.0 ± 1.6 g; and G3= -4.1 ± 1.5 g. There was a significant (*p* < 0.001) effect of body mass change over three time points (day 0, day 4, and day 9 post DOX) without a significant time*group interaction (*p* = 0.906).

#### Echocardiography results

**Fig. 3:**
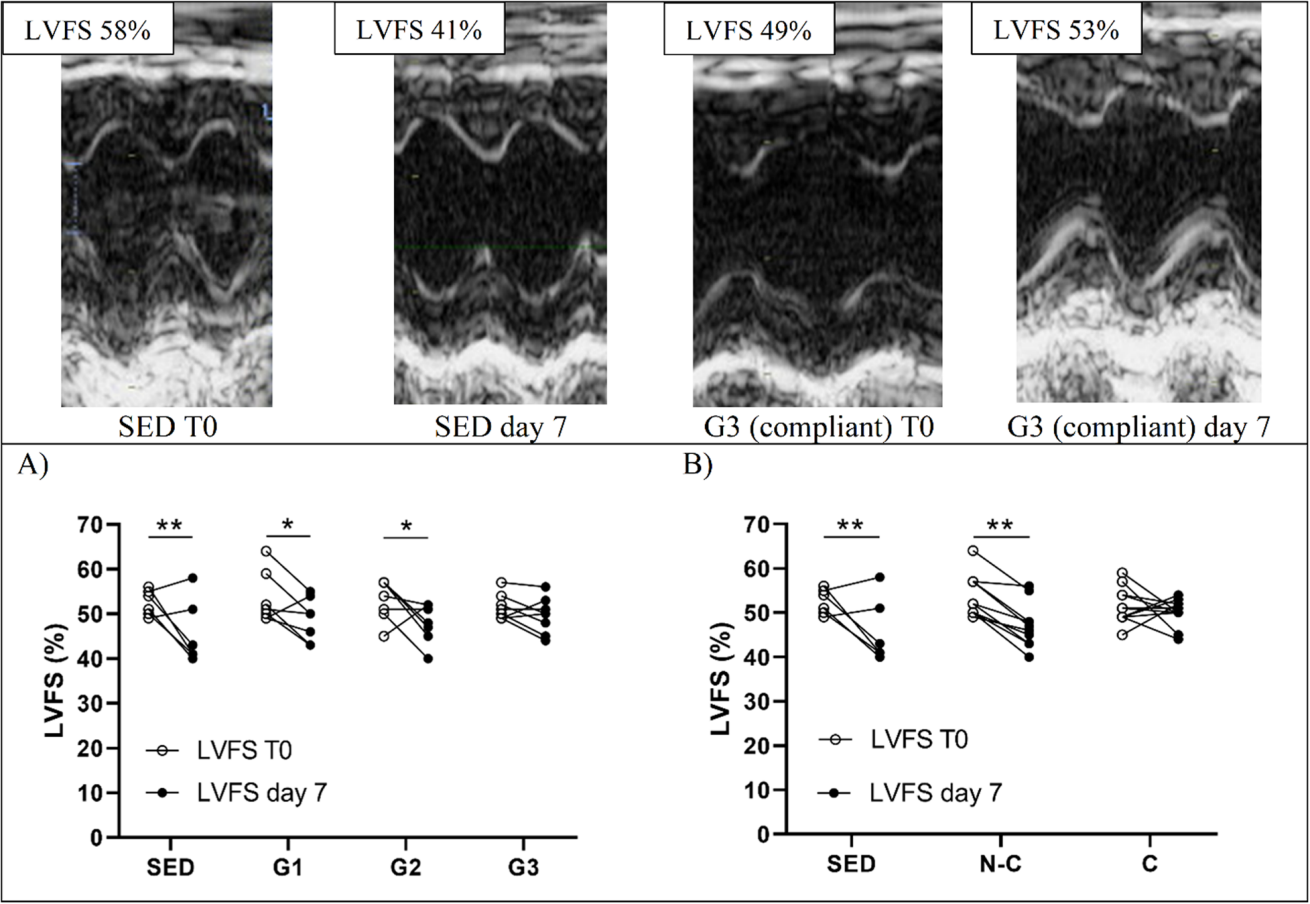
Change pre- and post-doxorubicin in left ventricular fractional shortening (**A**) according to intervention groups, there is a significant time effect (*p* < 0.001); (**B**) according to exercise compliance, there is a significant (*p* = 0.045) time*compliance interaction and a significant time effect (*p* < 0.001). *P < 0.05, **P < 0.01, represents Bonferroni corrected post hoc for time effect per group. Each circle represents a mouse. Representative M-mode images are displayed. C, compliant (*n* = 12); G1, HIIE day 1 post-DOX (*n* = 7); G2, HIIE day 2 post-DOX (*n* = 7); G3, HIIE day 3 post-DOX (*n* = 8); LVFS, left ventricular fractional shortening; N-C, non-compliant (*n* = 10) and SED, no exercise (*n* = 6)

Echocardiography parameters are presented in Table 1 and supplementary file 3 (LVmass). DOX treatment resulted in an overall posterior (*p* = 0.023) and septal (*p* = 0.004) wall atrophy, and a end-systolic dilatation (*p* = 0.047) compared to baseline values. Figure 3A shows LVFS changes pre- and post-DOX according to groups. Group analysis of pre- and post-DOX LVFS changes showed a significant LVFS reduction for SED (*p* = 0.009), G1 (*p* = 0.043), and G2 (*p* = 0.043), but none for the G3 group (*p* = 0.406). The SED group had the greatest reduction in LVFS (MΔ= -6.8 ± 7.5; 95% CI [-12.00; -1.33]) that represented a very large pre- and post-DOX effect size (*d* = 2.37). Among the exercise intervention groups, the G3 group had the smallest reduction in LVFS (MΔ=-1.7 ± 3.3; 95% CI [-4.00; 1.10]) with a medium effect size (*d* = 0.61) while G1 and G2 groups experienced larger reductions (MΔ=-4.7 ± 5.3; 95% CI [-7.57; -1.61] and MΔ=-5.1 ± 7.0; 95% CI [-10.70; 2.56], respectively), with larger effect sizes (*d* = 0.81 and *d* = 1.12, respectively). Overall, there was a significant reduction in LVFS (*p* < 0.001) post DOX but no significant time*group interaction (*p* = 0.438) despite the observed differences in effect size described above.

**Table 1:** Echocardiographic data at baseline and 7 days after doxorubicin treatment

	Analyses according to intervention group			Analyses according to exercise compliance		
	Group	Baseline (mean ± SD)	Day 7 (mean ± SD)	Group	Baseline (mean ± SD)	Day 7 (mean ± SD)
HR, beats/min	SED (*n* = 6) G1 (*n* = 7) G2 (*n* = 7) G3 (*n* = 8)	606 ± 86 643 ± 33 597 ± 34 586 ± 78	595 ± 43 581 ± 34^*^ 607 ± 49 598 ± 54	SED (*n* = 6) N-C (*n* = 10) C (*n* = 12)	606 ± 86 614 ± 41 602 ± 69	595 ± 43 570 ± 29^* †^ 617 ± 47^†^
SWT, mm	SED (*n* = 6) G1 (*n* = 7) G2 (*n* = 7) G3 (*n* = 8)	0.767 ± 0.082 0.743 ± 0.053 0.800 ± 0.058 0.750 ± 0.053	0.717 ± 0.041 0.757 ± 0.079 0.700 ± 0.058^**^ 0.687 ± 0.035^*^	SED (*n* = 6) N-C (*n* = 10) C (*n* = 12)	0.767 ± 0.082 0.760 ± 0.052 0.767 ± 0.065	0.717 ± 0.041 0.710 ± 0.088 0.717 ± 0.039
PWT, mm	SED (*n* = 6) G1 (*n* = 7) G2 (*n* = 7) G3 (*n* = 8)	0.750 ± 0.084 0.743 ± 0.053 0.743 ± 0.053 0.763 ± 0.106	0.717 ± 0.075 0.729 ± 0.095 0.657 ± 0.079 0.687 ± 0.064	SED (*n* = 6) N-C (*n* = 10) C (*n* = 12)	0.750 ± 0.084 0.770 ± 0.067 0.733 ± 0.078	0.717 ± 0.075 0.700 ± 0.082 0.683 ± 0.083
LVDd, mm	SED (*n* = 6) G1 (*n* = 7) G2 (*n* = 7) G3 (*n* = 8)	3.233 ± 0.413 3.257 ± 0.282 3.271 ± 0.386 3.200 ± 0.214	3.183 ± 0.172 3.186 ± 0.261 3.143 ± 0.190 3.163 ± 0.233	SED (*n* = 6) N-C (*n* = 10) C (*n* = 12)	3.233 ± 0.413 3.210 ± 0.288 3.267 ± 0.293	3.183 ± 0.172 3.180 ± 0.225 3.150 ± 0.224
LVDs, mm	SED (*n* = 6) G1 (*n* = 7) G2 (*n* = 7) G3 (*n* = 8)	1.550 ± 0.217 1.500 ± 0.258 1.557 ± 0.299 1.550 ± 0.160	1.717 ± 0.293 1.643 ± 0.282 1.643 ± 0.172 1.625 ± 0.103	SED (*n* = 6) N-C (*n* = 10) C (*n* = 12)	1.550 ± 0.217 1.480 ± 0.230 1.583 ± 0.233	1.717 ± 0.293 1.710 ± 0.218^**^ 1.575 ± 0.136
LVFS, %	SED (*n* = 6) G1 (*n* = 7) G2 (*n* = 7) G3 (*n* = 8)	52 ± 3 53 ± 6 53 ± 5 51 ± 3	46 ± 7^**^ 49 ± 5^*^ 48 ± 4^*^ 50 ± 4	SED (*n* = 6) N-C (*n* = 10) C (*n* = 12)	52 ± 3 54 ± 5 51 ± 4	46 ± 7^**^ 47 ± 5^**^ 50 ± 3

C, compliant; G1, HIIE day 1 post-DOX; G2, HIIE day 2 post-DOX and G3, HIIE day 3 post-DOX; HR, heart rate (mice HR at the time of echocardiographic imaging and measurement of cardiac dimensions); LVDd, left ventricle end-diastolic diameter; LVDs, left ventricle end-systolic diameter; LVFS, left ventricular fractional shortening; N-C, non-compliant; PWT, posterior wall thickness; SED, no exercise; SWT, septal wall thickness.*P < 0.05, **P < 0.01, represents Bonferroni corrected post hoc for time effect within each group; ^†^*P* < 0.05, represents differences between groups at either baseline or day 7.

#### Morphological and histological results

**Fig. 4:**
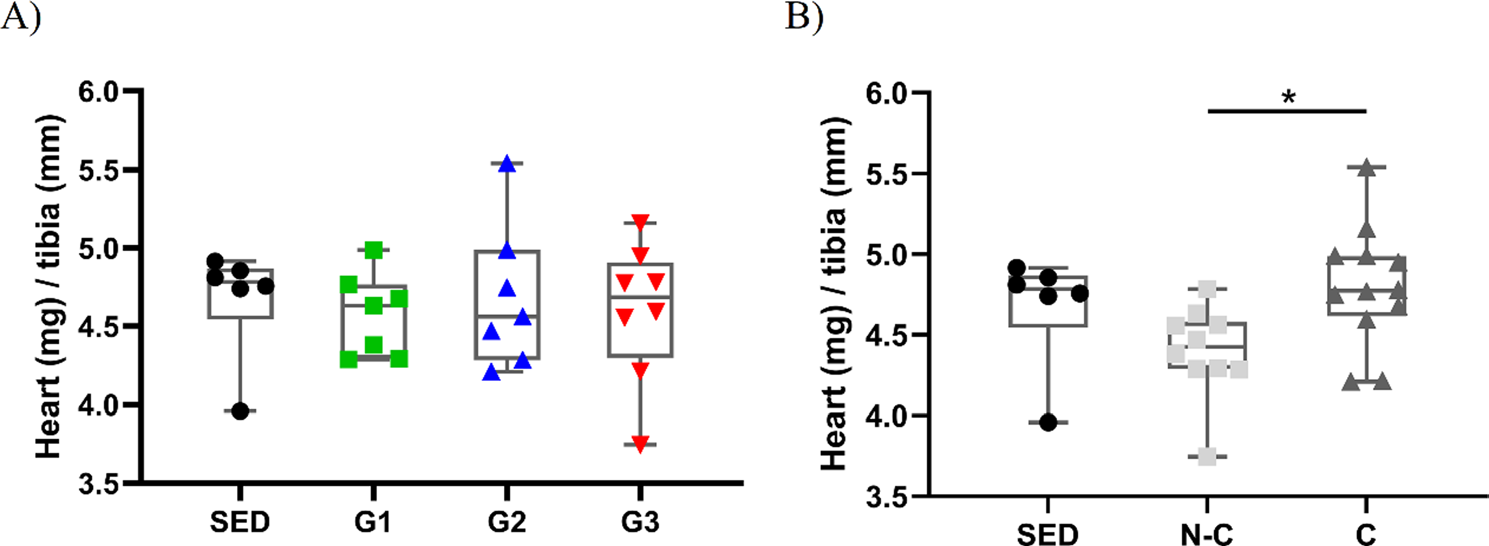
Heart mass to tibia length ratio (**A**) according to intervention groups there is no significant difference between groups. (**B**) Heart mass to tibia length ratio (mg/mm) according to exercise compliance groups; There is a significant difference (*p* = 0.029), *P < 0.05, represents Dunn-Bonferroni post hoc test. Values are median and interquartile. C, compliant; G1, HIIE day 1 post-DOX; G2, HIIE day 2 post-DOX; G3, HIIE day 3 post-DOX; N-C, non-compliant and SED, no exercise

**Fig. 5:**
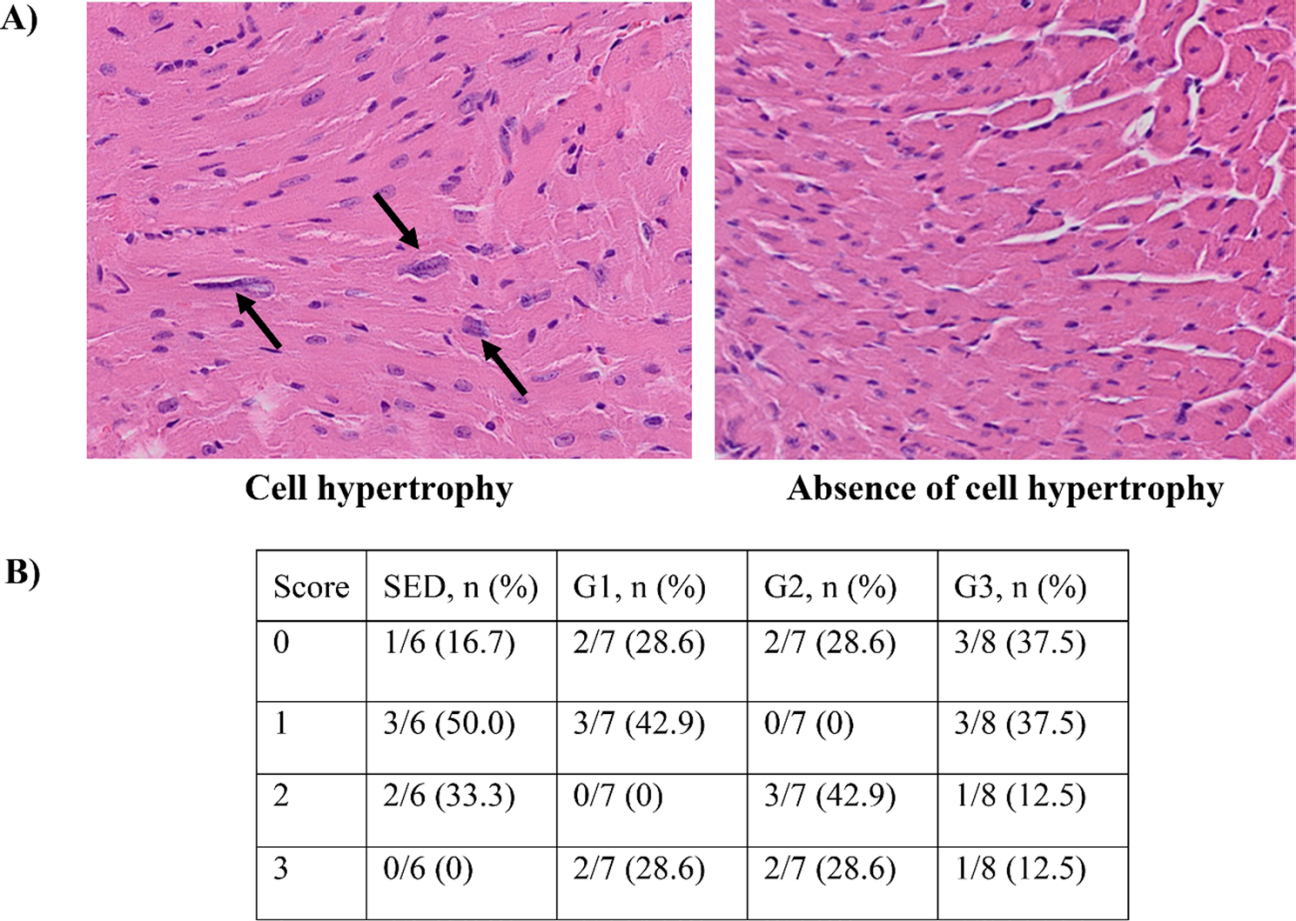
Cardiac cell hypertrophy (**A**) representative images magnified 200x; arrows represent hypertrophied cells. (**B**) Table representing quantification of hypertrophy scores at 9 days after doxorubicin treatment. Hypertrophy score 0, absence of cell hypertrophy; 1 ≤ 25%; 2, 25–50%; and 3 > 50% of cell hypertrophy. G1, HIIE day 1 post-DOX; G2, HIIE day 2 post-DOX; G3, HIIE day 3 post-DOX; and SED, no exercise. No significant difference between groups

Heart to tibia ratio did not differ significantly across groups (*p* = 0.885) as shown in Fig. 4A. No signs of necrosis, vacuolisation, inflammation, cytoplasmic clearing, or fibrosis were found in the histopathological analysis by two independent pathologists. Hypertrophy scores according to intervention groups and representative images are presented in Fig. 5 with no significant difference between groups (*p* = 0.366).

### HIIE effect according to exercise compliance

We observed that 10 of the 22 animals within the intervention groups (45%) were unable to complete the training (performing less than or equal to 50% of prescribed high-intensity bouts). We decided to analyse them separately and compared them to compliant (*n* = 12) and sedentary (*n* = 6) animals.

#### Effect on body mass

There was a significant (*p* < 0.001) body mass change over three time points (day 0, day 4 and day 9 post DOX), no difference between groups and a significant body mass change*compliance effect (*p* = 0.018) as depicted in Fig. 2B.

#### Echocardiography results

Echocardiography parameters are presented in Table 1 and pre- and post-DOX change in LVFS according to exercise compliance are depicted in Fig. 3B. SED and non-compliant (N-C) groups had a similar reduction in LVFS, MΔ= -6.8 ± 7.5; 96% CI [-12.00; -1.33] and MΔ= -6.7 ± 3.2; 95% CI [-9.00; -4.05] respectively, representing both a very large effect size (*d* = 2.37 and *d* = 1.39, respectively). For the compliant group (C), the decrease in LVFS was MΔ= -1.3 ± 5.6; 95% CI [-4.76; 1.92], representing a small effect size (*d* = 0.34). There was a significant (*p* < 0.001) time effect of LVFS change between pre- and post-DOX and a significant (*p* = 0.045) time*compliance interaction for LVFS. SED and N-C had a significant reduction in LVFS after DOX (*p* = 0.004 and *p* = 0.001 respectively), whereas no significant pre- and post-DOX difference was found for the C group (*p* = 0.396). Significant correlation was found between the number of high-intensity intervals completed and LVFS change (*p* = 0.031; R^2^ = 0.167; see Figure S2.2 in supplementary file 2).

#### Morphological and histological results

There was a significant difference between groups analysed according to compliance (C, N-C and SED) regarding heart to tibia ratio (*p* = 0.029). Post-hoc analysis showed that compliant animals had significantly larger normalized heart mass compared to non-compliant animals (*p* = 0.032, Fig. 4B). No statistically significant correlation was found between the number of high-intensity intervals completed and heart to tibia ratio (*p* = 0.079; R^2^ = 0.114), see Figure S2.3 in supplementary file 2. Hypertrophy scores based on compliance are presented in Table S2.1 in the supplementary file 2 and no significant difference (*p* = 0.729) was found between groups.

## Discussion

To our knowledge, this is the first study investigating the effect of HIIE performed shortly after DOX treatment, much less the effect of exercise timing relative to treatment administration. Our results show that one HIIE session following DOX treatment was feasible for C57bl/6 female mice and did not exacerbate cardiotoxicity in comparison with sedentary mice. In fact, an exercise session performed 3 days after DOX and, more importantly, a high level of compliance to the HIIE session were two factors which positively influenced the impact of DOX on LVFS.

### Feasibility of HIIE in a DOX treated animal model

More than half (54%) of our study animals were able to complete the 40-minutes HIIE session despite the highly cardiotoxic dose. All animals were encouraged to keep running to the point where they were unable to continue regardless of repeated stimulation. Pushing the animal to continue further despite obvious signs of difficulty or exhaustion could have resulted in deleterious effects. For example, a study using a 6 h swimming bout in rats showed acute deleterious effects such as increases in oxidative damage to tissues caused by increases in ROS and reduced redox antioxidant function [33].

The acclimatation protocol used in the study was short in duration (12 min) and of low to moderate intensity to limit the cardioprotective preconditioning effect related to longer exercise sessions performed at moderate to vigorous intensity. It is possible that an acclimatation protocol including intervals of moderate intensity could have better prepared the mice for HIIT and therefore increased compliance to the intervention.

Compliance to exercise in our study was similar at day 1, day 2 and day 3 following DOX. Non-compliance to exercise or reduced exercise capacity, as demonstrated in other studies following DOX, may be related to the treatment’s non-cardiac side effects such as asthenia, diarrhea and anorexia [34] affecting the animal’s physical capacity and willingness to exercise. Previous studies reported significant reduction in voluntary wheel running, reduced peak speed and a 40% decline in running performance following cardiotoxic treatment compared to non-treated animals [34–36]. This DOX induced reduction in exercise capacity may explain why 45% of our study’s animals were unable to perform all of the prescribed high-intensity bouts. Accordingly, Elsea et al.’s study [34], showed that animals tend to drastically reduce their voluntary wheel running activity on the day of the treatment followed by a gradual increase of their activity level over a period of 1–10 days. Therefore, we can hypothesize that tolerance and compliance to HIIE in these animals could be greater in the future if performed later than during the first 3 days after treatment.

### Effect of HIIE performed shortly following DOX treatment on cardiac function

We observed that animals unable to perform the HIIE session (non-compliant) had a similar and significant reduction in LVFS compared to sedentary animals, whereas LVFS did not change in compliant animals. To our knowledge no other study investigated the acute effect of a single exercise session performed immediately following DOX treatment though, some studies demonstrated that one moderate or vigorous exercise session performed 24 h hours prior to DOX treatment preserved cardiac function in rats and humans compared to their sedentary counterparts [13, 37]. Cardioprotective effects of acute vigorous efforts such as HIIE could be related to the systemic transient upregulation of total antioxidant capacity and anti-inflammatory effect (increases in white blood cells) that have been demonstrated in healthy humans following this type of exercise [38]. Therefore, one exercise session may have short term cardioprotective effect and performing HIIE following treatment (when feasible) may help preserve cardiac function.

Furthermore, previous studies demonstrated that chronic moderate intensity continuous exercise performed between DOX treatment cycles in C57bl/6 mice had cardioprotective effects by preserving LV end-systolic volume, LV internal dimension and strain rate, but did not find a significant effect on LVFS [36, 39]. Higher intensity exercise usually generates greater improvements in VO_2max_ and stroke volume than lower intensity exercise [17, 40]. Therefore, future studies should investigate the effect of multiple exercise sessions of HIIE between DOX treatment cycles in a chronic treatment administration regimen which may provide a greater cardioprotective effect related to training induced adaptations.

### Effect of HIIE performed concomitantly with DOX treatment on cardiac structure

Previous results from magnetic resonance imaging demonstrated that cardiac atrophy occurs shortly following anthracycline exposure in humans and is associated with worse clinical outcomes [41, 42]. In our study, one HIIE session did not influence normalized heart mass since no differences were found between exercising and sedentary animals regardless of exercise compliance. Since other studies reported that low to moderate-intensity chronic exercise partially preserved normalized heart mass and cardiomyocyte density [36, 39], we can hypothesize that only repeated HIIE sessions could influence DOX induced cardiac atrophy.

In our short-term high DOX dose animal model, no signs of necrosis, cytoplasmic clearing, vacuolisation, fibrosis or inflammation were detected at 9 days following bolus DOX injection. However, more than 25% of LV cardiac cells were hypertrophied in 39.3% of the total sample, which is consistent with Diaz et al’s study that found important increases in hypertrophic markers such as MYH7 and BNP, 7 days following a bolus DOX dose of 10 mg/kg. After one HIIE session, we did not observe sub-acute effect on cardiac cell hypertrophy. The long-term effect of HIIE combined with DOX on cardiac cell hypertrophy is unknown. Future studies should investigate the effect of HIIE training on pathological DOX-induced cardiac remodeling using a chronic exercise and DOX administration model with long-term study endpoints.

### Timing of HIIE relative to anthracycline treatment

While most animal studies investigated the cardioprotective effect of aerobic exercise interventions performed prior to treatment [12, 43], our study explored the effect of HIIE performed shortly after DOX treatment. Though exercise preconditioning has a greater cardiac function (LVFS) protective efficacy compared to concomitant exercise [12], performing exercise during treatment could confer additional benefits related to the effect of the treatment on the cancer itself. Two pre-clinical studies using xenograft murine tumor models demonstrated that exercise does not seem to attenuate the antitumor effect of DOX and may even enhance treatment effect and reduce tumor burden [44, 45]. Exercise intervention in these two previous studies was low intensity treadmill running or free wheel running. The effect of HIIT on tumor and treatment efficiency is still unknown. The next steps could be to investigate the effect of HIIT in a cancer murine model receiving anthracycline. The ideal moment to start exercising following DOX intervention needs to be further explored. However, our study suggests that some delay between the end of the infusion and the HIIE session might help to maximize its benefit on cardiac function.

### Study limitations

Some animals were unable to complete the exercise intervention which contributed to a loss of statistical power. Although we performed a more robust analysis (bootstrapped CI) and calculated effect sizes that are less dependent on sample size, our pilot results must be taken with cautious considering the size of our mice sample. Moreover, we cannot extrapolate this study’s results to male murine models or senescent animal models considering that sex hormones can offer a cardioprotective effect and no interventions were applied to control hormonal status such as using an ovariectomized animal model.

Also, it is to be noted that this study is a physiological model investigating the effect of exercise on DOX-induced cardiotoxicity without cancer and therefore limits its translation to the cancer care clinical context. Studies investigating acute exercise following DOX are rare, especially at high exercise intensity. This study was therefore designed as a first step in the investigation of the HIIE effect when performed shortly following treatment to inform future larger scale studies on the subject using chronic exercise intervention in a murine cancer model.

## Conclusions

This pilot animal-based study revealed that one single HIIE session performed shortly after DOX treatment is feasible in an animal model and did not exacerbate cardiotoxicity acutely. The time between the DOX infusion and the HIIE session as well as the level of compliance to exercise are two factors which could influence the negative impact of DOX on cardiac function. The long-term safety and potential benefits of multiple HIIE sessions performed shortly after DOX are still unknown and future studies are required to investigate the impact of repetitive HIIE sessions in a tumor-bearing animal model receiving multiple doses of DOX over a few weeks.

### Electronic supplementary material

Below is the link to the electronic supplementary material.

Supplementary Material 1

Supplementary Material 2

Supplementary Material 3

Supplementary Material 4

## Data Availability

The datasets used and/or analysed during the current study are available from the corresponding author on reasonable request.

## Abbreviations

DOX: doxorubicin
LV: left ventricular
ROS: reactive oxygen species
HIIT: high-intensity interval training (multiple exercise sessions over a given period)
HIIE: high-intensity interval exercise (one exercise session)
LVFS: left ventricular fractional shortening
Vpeak: peak running speed
