# Supplementary material for: Effect of acute high-intensity interval exercise on a mouse model of doxorubicin-induced cardiotoxicity: a pilot study
Source: BMC Sports Sci Med Rehabil. 2024 Apr 26;16:95. doi: 10.1186/s13102-024-00881-x (PMC11046902; doi:10.1186/s13102-024-00881-x)
Supplement: Supplementary file 1 — Supplementary Material 1 [file 13102_2024_881_MOESM1_ESM.docx]

**Supplementary file 1 - Pilot study to determine the experimental model**

Pilot study of female C57bl/6 “retired breeders” compared to male “retired breeders” (approximately 30-40 weeks old) receiving a single dose of either 4 mg/kg or 20 mg/kg of doxorubicin (DOX). Three groups: controls (CTRL) received a saline injection (n=2 male and n=2 female); DOX 4 mg/kg, received a low bolus intravenous DOX injection of 4 mg/kg (n=3 male and n=3 female) and DOX 20 mg/kg, received a high bolus intravenous DOX injection (n=3 male and n=3 female). All animals were sacrificed 7 days following injection.

**Figure S1.1 – Body mass following a high doxorubicin bolus injection (20 mg/kg) according to sex**

There is a significant time (*p*=0.001) effect on weight loss but no significant time*SEX interaction (*p*=0.096) using a repeated measure ANOVA. Bonferroni corrected post hoc analysis showed a significant difference between T0 and T5 for male mice (p=0.032) but not for female mice (*p*=0.308). T7 was significantly different than T0 for both sexes (female *p*=0.013; male *p*=0.002). T0, baseline before DOX injection; T5, 5 days after DOX injection; T7, 7 days after DOX injection.

**Figure S1.2 – Heart mass to tibia length ratio according to sex and DOX dose (low/high).**

Sex (*p*=0.043) and DOX dose (*p*=0.015) had a significant effect on heart to tibia ratio but there was no significant interaction (sex*dose; p=0.296) using a multi-factor ANOVA. Bonferroni corrected post hoc analysis showed a significant difference between CTRL animals and high DOX dose 20 mg/kg (*p*=0.026) but not compared to low DOX dose 4 mg/kg (*p*=0.347).

**Table S1.1 - pilot study result summary (pros and cons) for experimental model choice**

| Female C57bl/6 | Male C57bl/6 |
| --- | --- |
| Pros:   - Less lethargic than males despite high DOX dose - Lower normalized heart mass to tibia length ratio compared to males (marker of cardiotoxicity) | Pros: none |
| Cons:   - none | Cons:   - Mice possibly too lethargic to exercise following high DOX dose - Weight loss is greater and occurs sooner than females. Higher risk of reaching the study’s intervention point before the endpoint (body mass loss ≥20%) - Need to be caged individually (increased stress related to isolation) |

| Low bolus dose 4 mg/kg | High bolus dose 20 mg/kg |
| --- | --- |
| Pros:   - Less lethargic - Lower risk of reaching the study's intervention point (body mass loss ≥20%) before the endpoint | Pros:   - Significantly greater toxicity compared to CTRL (heart to tibia ratio) - Only one injection needed to reach significant cardiotoxicity (heart mass loss) |
| Cons:   - Lower toxicity (no difference on heart to tibia ratio, one of the study’s main outcomes) | Cons:   - Risk of reaching the study’s intervention point before endpoint |
