# Supplementary material for: Effect of acute high-intensity interval exercise on a mouse model of doxorubicin-induced cardiotoxicity: a pilot study
Source: BMC Sports Sci Med Rehabil. 2024 Apr 26;16:95. doi: 10.1186/s13102-024-00881-x (PMC11046902; doi:10.1186/s13102-024-00881-x)
Supplement: Supplementary file 2 — Supplementary Material 2 [file 13102_2024_881_MOESM2_ESM.docx]

**Supplementary file 2 – Additional compliance analyses**

**Figure S2.1 – Number of intervals completed (maximum of 16 bouts) according to exercise group training either on day 1 (G1), day 2 (G2) or day 3 (G3) after DOX injection.**


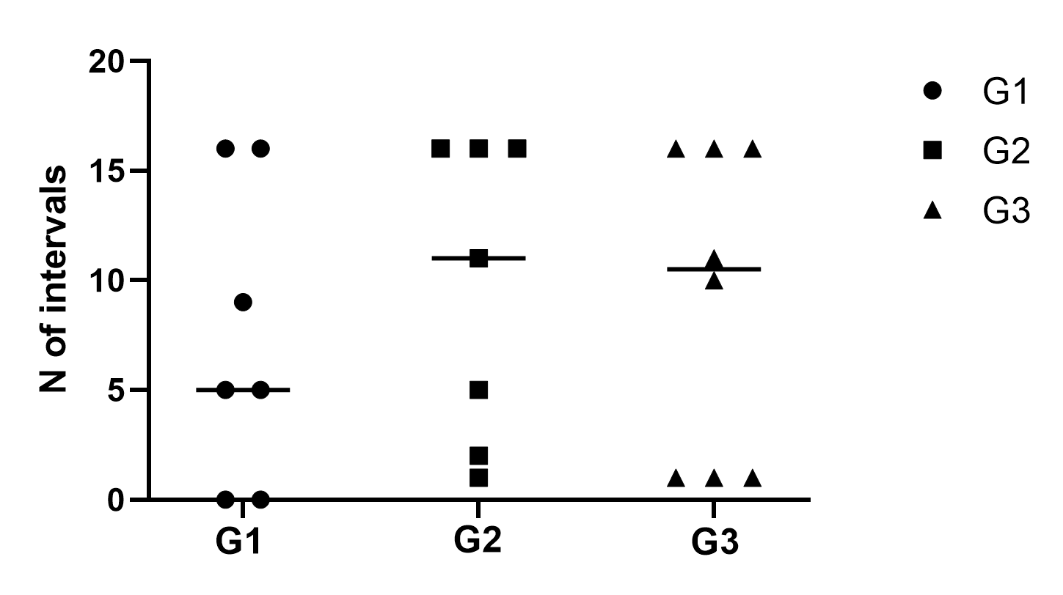


No significant difference between groups. Each point represents a mouse. G1, HIIE day 1 post-DOX (n=7); G2, HIIE day 2 post-DOX (n=7); G3, HIIE day 3 post-DOX (n=8).

**Figure S2.2 – Correlation plot representing left ventricular fractional shortening change (delta) according to the number of high-intensity intervals completed. Each dot represents a mouse color coded according to group.**


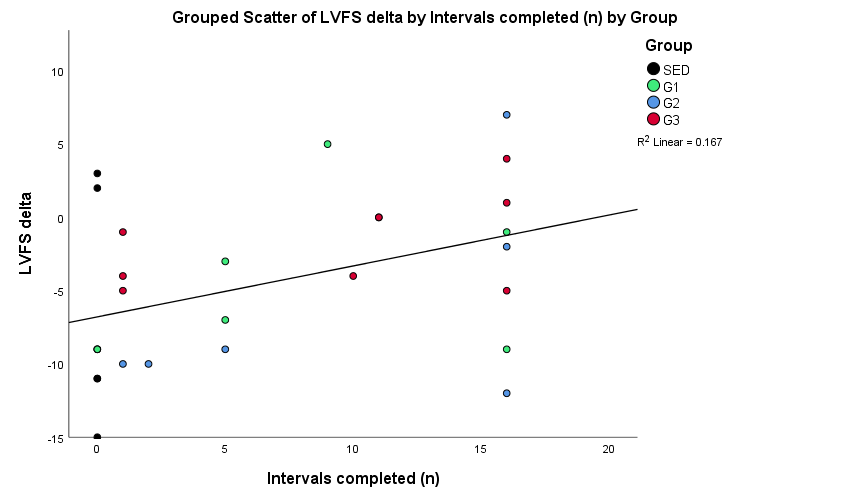


**Figure S2.3 – Correlation plot representing heart mass to tibia ratio according to the number of high-intensity intervals completed. Each dot represents a mouse color coded according to group.**


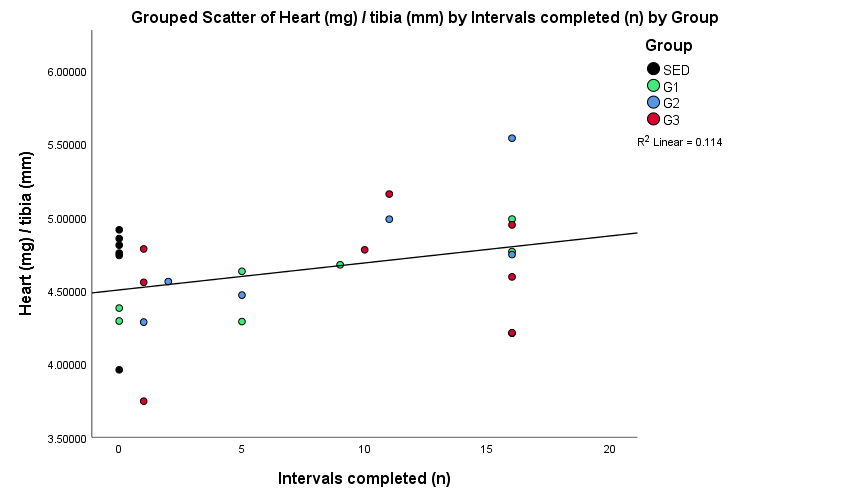


Statistically significant correlation was detected between the number of high intensity intervals completed and left ventricular fractional shortening (LVFS) change (*p*=0.031) but not for heart to tibia ratio (*p*=0.079). The goodness of fit of the models were low with R^2^=0.167 and R^2^=0.114 for LVFS delta and heart to tibia ratio respectively. There seems to be a relationship between the number of high-intensity intervals performed and the reduction in LVFS, where animals able to complete more intervals have smaller DOX-induced LVFS reduction.

**Table S2.1 – Cardiac cell hypertrophy scores at 9 days after doxorubicin treatment according to compliance.**

| **Score** | **SED, n (%)** | **N-C, n (%)** | **C, n (%)** |
| --- | --- | --- | --- |
| **0** | 1/6 (16.7) | 4/10 (40.0) | 3/12 (25.0) |
| **1** | 3/6 (50.0) | 3/10 (30.0) | 3/12 (25.0) |
| **2** | 2/6 (33.3) | 1/10 (10.0) | 3/12 (25.0) |
| **3** | 0/6 (0) | 2/10 (20.0) | 3/12 (25.0) |

Hypertrophy score 0, absence of cell hypertrophy; 1 ≤ 25%; 2, 25-50%; and 3 > 50% of cell hypertrophy. C, compliant; N-C, non-compliant and SED, no exercise. No significant difference between groups.
