# Supplementary material for: Effect of acute high-intensity interval exercise on a mouse model of doxorubicin-induced cardiotoxicity: a pilot study
Source: BMC Sports Sci Med Rehabil. 2024 Apr 26;16:95. doi: 10.1186/s13102-024-00881-x (PMC11046902; doi:10.1186/s13102-024-00881-x)
Supplement: Supplementary file 4 — Supplementary Material 4 [file 13102_2024_881_MOESM4_ESM.docx]

**Supplementary file 3 - Analysis of left ventricular (LV) mass**

LVmass was estimated from echocardiography measurements in short axis M-mode.

LV mass calculated using Penn’s formula as described in Stypmann et al.’s article (1):
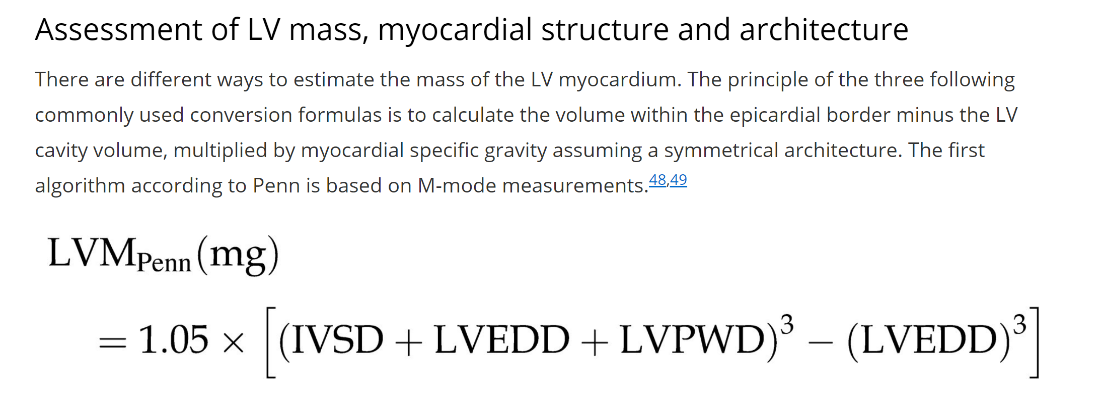


LVmass to tibia ratio was calculated:

LVmass (mg) / tibia length (mm)

**Analysis of left ventricular (LV) mass pre- and post-DOX according to intervention groups**

**Table S1.** Mean LVmass normalized to tibia length (mg/mm) according to intervention groups

| Group | Time | Mean | Std. Error | 95% Confidence Interval | |
| --- | --- | --- | --- | --- | --- |
|  |  |  |  | Lower Bound | Upper Bound |
| Sedentary  (n=6) | 1 | 4.428 | 0.258 | 3.895 | 4.961 |
|  | 2 | 4.008 | 0.266 | 3.459 | 4.556 |
| G1  (n=7) | 1 | 4.430 | 0.239 | 3.937 | 4.924 |
|  | 2 | 4.293 | 0.246 | 3.785 | 4.801 |
| G2  (n=7) | 1 | 4.710 | 0.239 | 4.217 | 5.204 |
|  | 2 | 3.708 | 0.246 | 3.200 | 4.216 |
| G3  (n=8) | 1 | 4.428 | 0.224 | 3.966 | 4.889 |
|  | 2 | 3.831 | 0.230 | 3.355 | 4.306 |


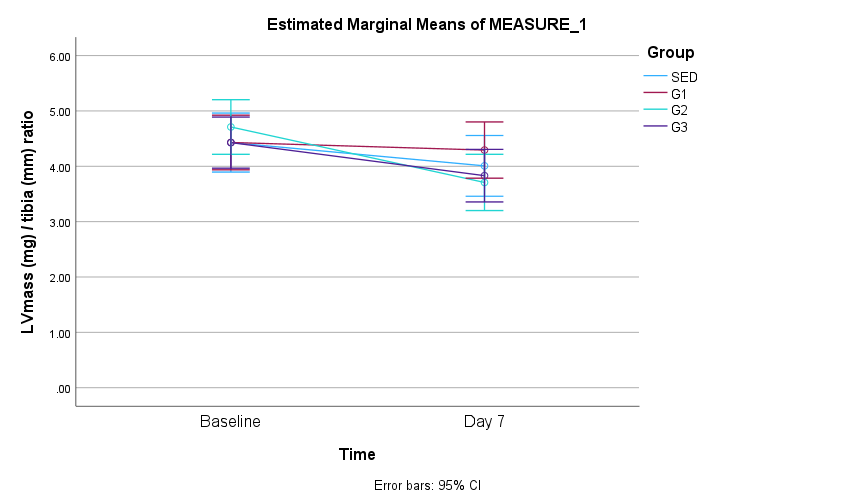


**Figure S1.** Estimated LVmass pre and post-DOX normalized to tibia length (mg/mm) according to intervention groups

There was a significant overall pre-post time effect (*p*< 0.007). No significant Time*Group interaction (*p*=0.413).

**Table S2.** Mean LVmass (mg) according to intervention groups

| Group | Time | Mean | Std. Error | 95% Confidence Interval | |
| --- | --- | --- | --- | --- | --- |
|  |  |  |  | Lower Bound | Upper Bound |
| Sedentary(n=6) | 1 | 76.716 | 4.487 | 67.455 | 85.978 |
|  | 2 | 69.511 | 4.488 | 60.250 | 78.773 |
| G1  (n=7) | 1 | 76.193 | 4.154 | 67.618 | 84.767 |
|  | 2 | 73.906 | 4.155 | 65.331 | 82.480 |
| G2  (n=7) | 1 | 80.627 | 4.154 | 72.053 | 89.202 |
|  | 2 | 63.186 | 4.155 | 54.611 | 71.761 |
| G3  (n=8) | 1 | 75.576 | 3.886 | 67.555 | 83.596 |
|  | 2 | 65.374 | 3.886 | 57.353 | 73.395 |


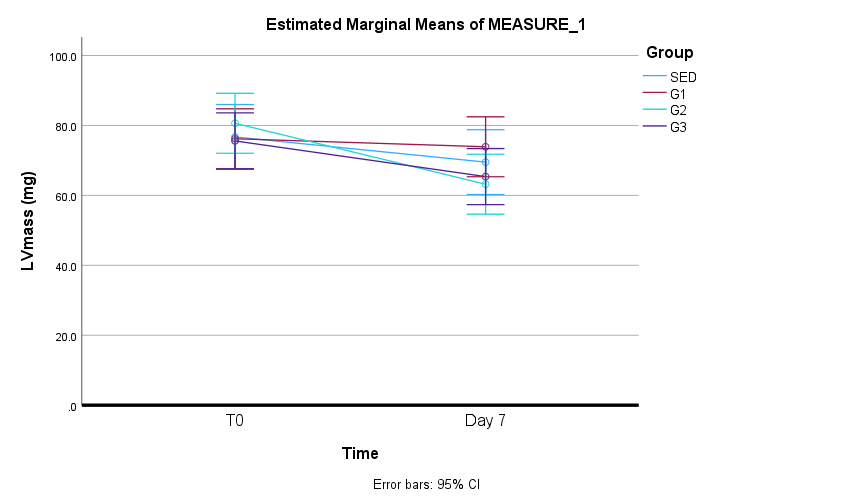


**Figure S2.** Estimated LVmass (mg) pre and post-DOX according to intervention groups

**Analysis of left ventricular (LV) mass pre- and post-DOX according to complianc**

**Table S3.** Mean LVmass normalized to tibia lenght (mg/mm) according to compliance

| Compliance status | Time | Mean | Std. Error | 95% Confidence Interval | |
| --- | --- | --- | --- | --- | --- |
|  |  |  |  | Lower Bound | Upper Bound |
| Sedentary  (n=6) | 1 | 4.428 | 0.258 | 3.898 | 4.959 |
|  | 2 | 4.008 | 0.277 | 3.438 | 4.578 |
| Non-compliant  (n=10) | 1 | 4.479 | 0.200 | 4.068 | 4.890 |
|  | 2 | 3.985 | 0.214 | 3.544 | 4.426 |
| Compliant  (n=12) | 1 | 4.551 | 0.182 | 4.176 | 4.927 |
|  | 2 | 3.900 | 0.196 | 3.497 | 4.303 |


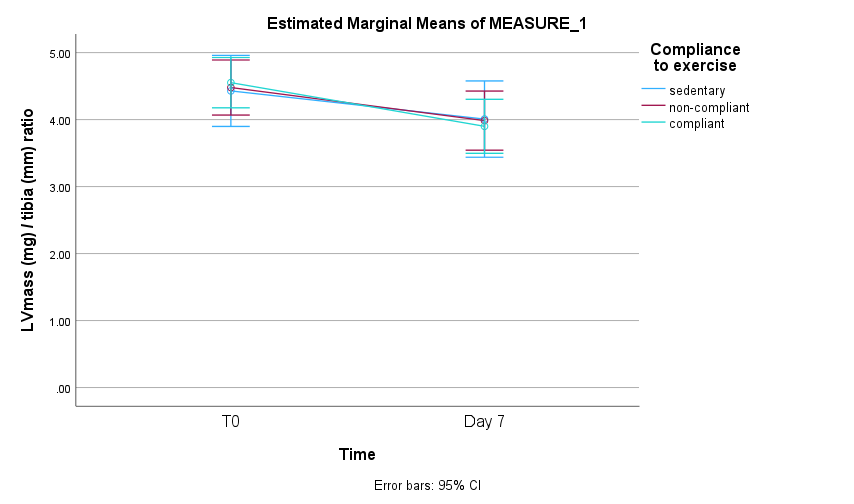


**Figure S3.** Estimated LVmass (mg) to tibia (mm) ratio pre and post-DOX according to compliance

There was a significant overall pre-post time effect p=0.013. No significant Time*Compliance interaction (p=0.879).

**Table S4.** Mean LVmass (mg) according to compliance

| Compliance to exercise | Time | Mean | Std. Error | 95% Confidence Interval | |
| --- | --- | --- | --- | --- | --- |
|  |  |  |  | Lower Bound | Upper Bound |
| Sedentary  (n=6) | 1 | 76.716 | 4.479 | 67.493 | 85.940 |
|  | 2 | 69.511 | 4.710 | 59.811 | 79.212 |
| Non-Compliant  (n=10) | 1 | 77.176 | 3.469 | 70.031 | 84.321 |
|  | 2 | 68.571 | 3.648 | 61.057 | 76.086 |
| Compliant  (n=12) | 1 | 77.549 | 3.167 | 71.027 | 84.071 |
|  | 2 | 66.410 | 3.331 | 59.550 | 73.269 |


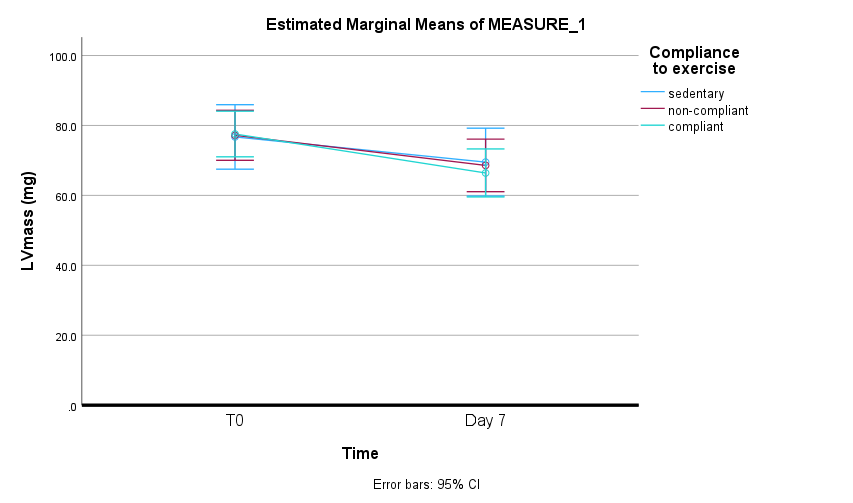


**Figure S4.** Estimated LVmass (mg) pre and post-DOX according to compliance

**References**

1. Stypmann J, Engelen MA, Troatz C, Rothenburger M, Eckardt L, Tiemann K. Echocardiographic assessment of global left ventricular function in mice. Lab Anim. 2009;43(2):127-37.
